# UK Biobank—A Unique Resource for Discovery and Translation Research on Genetics and Neurologic Disease

**DOI:** 10.1212/NXG.0000000000200226

**Published:** 2025-01-17

**Authors:** Hannah Taylor, Melissa Lewins, M. George B. Foody, Oliver Gray, Jelena Bešević, Megan C. Conroy, Rory Collins, Ben Lacey, Naomi Allen, Lucy Burkitt-Gray

**Affiliations:** 1 Oxford Population Health (Nuffield Department of Population Health), University of Oxford, United Kingdom; and; 2 UK Biobank, Stockport, Greater Manchester, United Kingdom.

## Abstract

UK Biobank is a large-scale prospective study with extensive genetic and phenotypic data from half a million adults. Participants, aged 40 to 69, were recruited from the general UK population between 2006 and 2010. During recruitment, participants completed questionnaires covering lifestyle and medical history, underwent physical measurements, and provided biological samples for long-term storage. Whole-cohort assays have been conducted, including biochemical markers, genotyping, whole-exome and whole-genome sequencing, as well as proteomics and metabolomics in large subsets of the cohort, with potential for additional assays in the future. Participants consented to link their data to electronic health records, enabling the identification of health outcomes over time. Research studies using UK Biobank data have already enhanced our understanding of the role of genetic variation in neurologic disease, offering insights into potential therapeutic approaches. The integration of genetic and imaging data has led to significant discoveries regarding the relationship between genetic variants and brain structure and function, particularly in Alzheimer disease and Parkinson disease. Genetic data have also allowed Mendelian randomization analyses to be performed, enabling further investigation into the causality of associations between behavioral and physiologic factors—such as diet and blood pressure—and neurologic outcomes. Furthermore, genetic and proteomic data have been particularly useful in identifying new drug targets for neurologic disease and in enhancing risk prediction algorithms that are increasingly applied in clinical practice to identify those at higher risk. As UK Biobank continues to be enhanced, and the cases of neurologic disease accrue over time, the study will become increasingly valuable for both discovery and translational research on genetics and neurologic disease.

## Introduction

Neurologic disease places a major burden on population health and health systems worldwide. In 2021, neurologic disease was the leading cause of global disability-adjusted life years, and the second leading cause of death.^1,2^ Over the past few decades, the number of individuals living with or dying from neurologic disorders (including Alzheimer disease, Parkinson disease, and stroke) has risen. This rise reflects, in part, global increases in population growth and age, and exposures to environmental, metabolic, and lifestyle risk factors which are particularly relevant for noncommunicable diseases, such as stroke and dementia. Yet, our understanding of the causes of many neurologic diseases, and the availability of effective treatments, remains limited.

Large-scale prospective cohort studies with follow-up over many years and deeply characterized assessment of exposures and disease outcomes offer the opportunity to further the understanding of the determinants of neurologic disease and identify potential targets for intervention. Large prospective studies, such as UK Biobank, allow assessment of a broad range of risk factors with a broad range of outcomes, and the scale of these studies allows associations to be assessed with precision (and, with a large sample size, permits the assessment of rarer disease). The long follow-up period enables correction for effects of reverse causality, which are particularly relevant for many neurologic diseases that have a long prodrome. Studies which have collected genetic data have also proven particularly valuable in the development of new drugs; recent studies have established that drugs with human genetic evidence are more than twice as likely to reach approval as those without.^3,4^

UK Biobank is a prospective cohort study of 500,000 adults, with collection of blood and other biological samples. Participants have been extensively phenotyped, and the original dataset has been expanded to include comprehensive imaging, genetics, and linkage to health records.^5^ As one of today's leading resources for health research, UK Biobank has been used by more than 8,000 research institutes worldwide and has produced more than 8,000 publications, to date. In this review, we describe the design of UK Biobank, the enhancements which have been made to the study, and the recent research on neurologic disease, with a particular focus on studies which have leveraged the extensive genetic data. In addition, we will describe how researchers can access the data for health-related research in the public interest.

## UK Biobank: Study Design

UK Biobank is a large-scale prospective cohort study of more than 500,000 adults. Established by Wellcome and the UK Medical Research Council, the study was designed to facilitate research into the genetic, lifestyle, and environmental determinants of disease, with the goal of advancing public health.^6^ Details of the study design have been described in detail elsewhere.^7^ Briefly, between 2006 and 2010, individuals registered with the National Health Service (NHS) aged between 40 and 69 years were invited to join the study. In total, 503,000 participants (54% female; median age 58 years) attended an initial baseline assessment in one of 22 bespoke centers distributed around the United Kingdom.^8^ Participants completed an electronic questionnaire covering factors relating to lifestyle, family history, local environment, employment, current or previous health conditions, medications, and operations (Figure). A trained nurse then conducted a computer-assisted interview to ensure accurate recording of diagnoses and medical events. Anthropometric measures (e.g., height, weight, and bioimpedance measures of body composition), and a range of other physical measures (e.g., grip strength, blood pressure, heart rate, and spirometry) and biological samples (blood, urine, and saliva) were also collected from participants. Further measurements were taken from a subset of the cohort, including retinal imaging (optical coherence tomography and fundus photography), intraocular pressure and refractive index, a calcaneal ultrasound to assess bone mineral density, and a cardiorespiratory fitness test (Table 1).^9^ Participants gave permission for UK Biobank to store their biological samples, to contact them regarding future follow-up opportunities, and to link to their health-related records over time.

**Figure:**
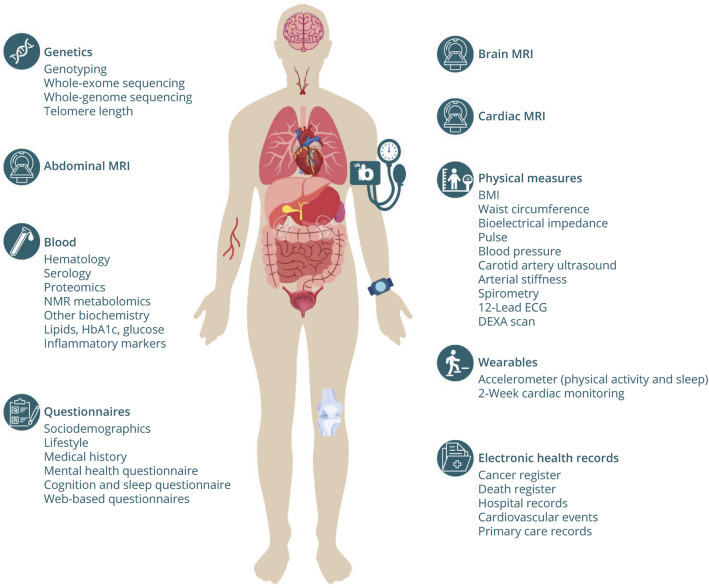
Outline of the Types of Data Available From UK Biobank

## Enhancements to UK Biobank

Since recruitment, UK Biobank has sought to enhance the characterization of exposures and health outcomes in the study. Soon after the baseline survey, between 2012 and 2013, a repeat of the baseline assessment was conducted in 20,000 participants to assess short-term variability in baseline measures and allow correction for regression dilution bias (the bias that results from the inaccuracy with which a single measurement of an exposure at baseline characterizes long-term average levels; Table 1). From 2013 to 2016, 100,000 UK Biobank participants wore triaxial wrist accelerometers for 7 days to obtain objective measurements of physical activity and sleep patterns; in 2019, a subset of approximately 2,500 participants subsequently wore the accelerometers again at various intervals throughout the year to assess seasonal variations in physical activity. In 2014, UK Biobank initiated the world's largest multimodal imaging study and is anticipated to complete the imaging of 100,000 participants during 2025. Participants were invited to one of 4 imaging centers across England to undergo a range of physical measures, including MRI of the brain, heart and body, electrocardiography, carotid ultrasound, and full body dual X-ray absorptiometry scanning (DXA).^6^ During these visits, participants also repeated the questionnaires, physical measures, and biological sample collection as at baseline. From 2019, participants have been invited to repeat the imaging assessment, with the aim to image 60,000 participants, further enriching the longitudinal aspect of the imaging study.

**Table 1:** UK Biobank Data: Baseline Questionnaire, Physical Measures, Imaging, and Wearables

Data type	Details	Number of participants	Date of acquisition	Year first available
Baseline questionnaire	Sociodemographic factors, family history, psychosocial factors, local environment, lifestyle, health status, medical history, cognitive function	Whole cohort (baseline)	2006–2010	2012
		20,000 (first resurvey)	2012–2013	2013
		100,000 target (imaging visit)	2014–	2014
		60,000 target (repeat imaging)	2019–	2019
Baseline physical measures	Blood pressure and heart rate, hand grip strength, anthropometry (including bioimpedance), spirometry, heel bone density, arterial stiffness, hearing, eye examination, ECG (at rest and during activity)	Whole cohort (baseline)	2006–2010	2012
		20,000 (first resurvey)	2012–2013	2013
		100,000 target (imaging visit)	2014–	2014
		60,000 target (repeat imaging)	2019–	2019
Imaging assessment	Abdominal, brain, and heart MRI; full-body DEXA; carotid ultrasound; ECG	100,000 target (imaging visit	2014–	2014
		60,000 target (repeat imaging)	2019–	2019
Physical activity monitor	Accelerometer data on duration and intensity of physical activity	100,000	2013–2016	2016
		2,500 (repeat measurements)	2018	2019
Cardiac monitoring	14 d continual ECG to assess atrial fibrillation	36,000 target	2022–	Pending

An additional enhancement to the resource includes linkage to electronic health records (EHRs), including national cancer and mortality data, hospital inpatient admissions, and primary care data, which enables the identification of participants with incident and prevalent disease (Table 2). Primary care data, which contain coded data on symptoms, diagnoses, and prescriptions, are particularly useful to identify disorders and diseases which are unlikely to result in hospitalization but nonetheless have high morbidity, such as diabetes, dementia, and mental health disorders. To date, primary care records up to 2016 are available for approximately half of the cohort, although plans are underway to extend this to the full cohort. Linkage has also been established to enhance information on geographical exposures. For example, data are available regarding air and noise pollution, alongside metrics of the natural and built environment. In addition, web-based questionnaires are regularly administered to all UK Biobank participants with a contact email address (∼330,000 participants), with the aim of identifying health outcomes that are not well captured in routine health records (such as sleep, pain, mental health, and cognitive function) and to collect more detailed information on exposures of interest to public health (such as occupational history, diet, and adverse life events; Table 3).

**Table 2:** Health Record Linkages in UK Biobank

Data type	Details	No. of participants	Date of acquisition	Year first available
Death registrations	ICD-coded cause-specific mortality	Whole cohort	2006–	2012
Cancer registrations	ICD-coded cancer diagnoses	Whole cohort	England 1971–	2012
			Scotland 1957–	
			Wales 1971–	
Hospital admissions	ICD-coded diagnoses, and OPCS-coded procedures, from hospital inpatient records, including critical care	Whole cohort	England 1997–	2012
			Scotland 1977–	
			Wales 1999–	
Primary care	Includes read-coded data on diagnoses, prescriptions, and referrals	230,000	England 1938–2016	2019
			Scotland 1939–2017	
			Wales 1948–2017	
SARS-CoV-2 antigen tests	Data on test result and date, and laboratory	Whole cohort	2020–	2020

**Table 3:** Web-Based Questionnaires in UK Biobank

Data type	Details	No. of participants	Date of acquisition	Year first available
Diet	A 24-h dietary recall questionnaire	210,000 (4 occasions)	2011	2012
Cognitive function	Several of the cognitive function tests administered via touchscreen during the initial assessment visit were reimplemented as web-based questionnaires. Includes Device and Mood, Fluid intelligence, Trail making, Symbol digit substitution, Pairs matching, Numeric Memory, Matrix completion	124,000	2014–2015	2015
		180,000	2021–2023	
Occupational history	Work history over lifetime	122,000	2015	2015
Mental health	Lifetime and current mental disorders and symptoms with a major focus on depression and anxiety. Includes questions related to traumatic events in childhood and adulthood	158,000	2017	2017
Digestive health	Phenotyping of digestive disorders	176,000	2017	2018
Food preferences	Biological and environmental factors that influence food preferences or lifestyle choices	174,000	2019	2020
Chronic pain	Type, severity, and duration of chronic pain and impact on quality of life	169,000	2019	2021
Mental well-being	Expansion of mental health questionnaire delivered in 2017	80,000	2022–2023	2023
Health and well-being	Possible symptoms and impacts of long COVID	196,000	2022–2023	2023
Sleep outcomes	Sleep quality, insomnia, and sleep disturbances	181,000	2023–2024	Pending
Social interaction and focus	Social interaction, quality of relationships, capacity to focus, and control impulsive behaviour	Pending	2023-	Pending
Visualization and memory	Recognition and recall of faces, facts, and events	Pending	2023-	Pending
Repeat chronic pain	Expansion of chronic pain question delivered in 2019	Pending	2024-	Pending

Biological samples collected from participants have been processed to generate genomic, proteomic, metabolomic, and other biomarker data (Table 4).^6^ The UK Biobank dataset now contains genotyping, whole-exome sequencing (WES), and whole-genome sequencing (WGS) for all participants, which provides an unprecedented opportunity for researchers to explore human genetic variation and its relevance for disease. In addition, data on blood-based proteomics are currently available for about 55,000 participants, with a view to scale this up over time.^10^ Through combining human genetics with large-scale proteomics, EHR, and imaging data, UK Biobank is helping to bridge the gap between the human genome and the pathologic mechanisms underlying human diseases.

**Table 4:** Genetic Measures and Blood-Based Biomarkers in UK Biobank

Data type	Details	No. of participants	Date of acquisition	Year first available
Genetic measures				
Genotype	Genome-wide genotyping was performed using the BiLEVE Axiom array (∼50,000 participants) and the UK Biobank Axiom array (∼450,000 participants). Approximately 850,000 variants were directly measured, with >90 million variants imputed using the Haplotype Reference Consortium and UK10K and 1,000 Genomes reference panels	Whole cohort (baseline)	2006–2010	2017
Whole-exome sequencing	Whole-exome sequencing was performed by Regeneron and GlaxoSmithKline (∼50,000 participants) and completed by a further consortium (comprising Regeneron, AbbVie, Alnylam Pharmaceuticals, AstraZeneca, Biogen, Pfizer, Takeda, and Bristol Myers Squibb)	Whole cohort (baseline)	2006–2010	2019
Whole-genome sequencing	Whole-genome sequencing was performed to complement and enhance the extant genotype and exome data. The Medical Research Council funded a pilot project to perform whole-genome sequencing on 50,000 participants, undertaken by the Wellcome Sanger Institute, Cambridge. Government (UK Research and Innovation [UKRI]), pharmaceutical (Amgen, AstraZeneca, GlaxoSmithKline, and Johnson & Johnson), and charity (Wellcome Trust) bodies funded whole-genome sequencing of the remaining 450,000 participants	Whole cohort (baseline)	2006–2010	2021
Telomere length	Telomere length has been derived for the majority of participants, subject to removal of duplicates and QC	Whole cohort (baseline)	2006–2010	2021
		20,000 (first resurvey)	2012–2013	
Biochemical measures				
Blood assay markers	Biomarkers assayed in plasma, serum, red blood cells, and urine samples; includes established risk factors for disease (e.g., lipids for vascular disease, sex hormones for cancer), diagnostic measures (e.g., HbA1c for diabetes and rheumatoid factor for arthritis), and additional measures (e.g., liver and renal function tests)	Whole cohort (baseline)	2006–2010	2016
		20,000 (first resurvey)	2012–2013	
Infectious agents	Measurement of antibody seropositivity status of 20 pathogens	10,000 (baseline)	2006–2010	2019
Plasma metabolites	Plasma concentrations of >200 circulating metabolites (predominantly lipids) were measured using the NMR metabolomics platform by Nightingale Health	Whole cohort (baseline)	2006–2010	2021
		20,000 (first resurvey)	2012–2013	
Plasma proteins	Proteomic profiling was performed on blood plasma samples using the antibody-based Olink Explore 3072 PEA, measuring 2,941 protein analytes and capturing 2,923 unique proteins	54,000 participants (baseline)	2006–2010	2023

## Neurology and Genetic Research in UK Biobank

The diverse UK Biobank data have substantial potential to further understanding into the genetic, physiologic, lifestyle, and environmental determinants of a broad range of neurologic diseases. In particular, the genomic data, in combination with longitudinal data from imaging, biomarkers, and health outcomes enable diverse methods for exploration of how changes in lifestyle and physiologic markers affect the development of a wide range of neurologic diseases. In the following sections, we review these analyses and approaches and identify areas with significant potential for innovative research into neurologic disorders. We also highlight and provide perspectives on longitudinal analyses which are being enabled by the continuing growth of the UK Biobank resource.

### Genetic Determinants of Neurologic Disease

Genome-wide association studies (GWAS) have been used extensively to identify genetic variants that are associated with particular phenotypic traits and diseases. These often combine UK Biobank with data from other studies to maximize statistical power.^6,11^ One GWAS, which combined data from UK Biobank with the CHARGE and COGENT consortia, found more than 400 independent single-nucleotide polymorphisms (SNPs) across 148 loci associated with cognitive function; 58 of these loci were novel and were associated with a range of conditions, including neurodegenerative and neurodevelopmental disorders, brain structure, and psychiatric illnesses.^12^ Another study which performed a GWAS for Parkinson disease using data from 17 cohorts, including UK Biobank, found 90 independent genome-wide significant SNPs, of which 38 were novel, and explained 16%–36% of the heritable risk.^13^ To date, most GWAS which have used UK Biobank data for neurologic research have focused mainly on dementia, although other studies have also identified variants for multisite chronic pain, myasthenia gravis, and cerebral small vessel disease.^14-16^

The genotyping data, used for GWAS, were complemented by the release of WES data. For example, a UK Biobank study that performed a GWAS followed by an exome-wide association study found evidence that mitochondrial DNA copy number is a potential causative risk factor for dementia.^17^ The release of WES data has been of particular value to the biopharmaceutical industry, which has increasingly used genetic evidence from large-scale biobanks to inform and improve success in drug discovery.^4^ Analyses of exome sequencing from 454,000 UK Biobank participants resulted in the identification of 12 million coding variants, with more than 500 being significantly associated with a very wide range of health-related traits. The size of the study allowed for rare variants associated with phenotypic traits to be identified of which more than 90% were independent of the more common genetic variant signals.^18^

The recent availability of WGS data for all UK Biobank participants provides an unprecedented opportunity to explore human genetic variation and its effect on human health and disease. WGS data have already provided additional insights to those provided by WES data, including the identification of additional coding variants within exons, alongside those present in noncoding regions of the genome which play a crucial role in regulating gene and protein expression.^18^ The opportunity to analyze sequences outside of coding regions will enable the identification of rare noncoding variants that may have large effects on disease risk (or more common variants that have smaller effects), which are likely to be very important for future drug discovery.

The power of UK Biobank's genetic data has been supported by open-access online tools which report the summary statistics derived from analyses of UK Biobank genetic data.^19-21^ The availability of online tools improves accessibility to the results of genetic analyses on UK Biobank data, ensuring that these are easily accessible to a wide range of researchers and clinicians.

### Case Study: *APOE* Genotype and Alzheimer Disease

Alzheimer disease is a common neurologic condition with strong evidence of heritability. Most genetic loci contribute only a small amount to an individual's risk of developing Alzheimer disease, with the exception of the Apolipoprotein-E4 (*APOE4*) genotype, which is responsible for a threefold increase in risk ratio.^22^ As a result, *APOE* has been studied extensively for its involvement in the development of Alzheimer disease and a range of other neurologic and non-neurological disorders.^23^ The extensive genetic characterization of UK Biobank participants, together with its large size and longitudinal follow-up of health outcomes, has enabled the study of *APOE4* in a much larger research context.^23-25^ UK Biobank includes more than 5,000 individuals who are homozygous for the *APOE*-ε4 allele, and research has quantified the extent to which heterozygosity and homozygosity of the *APOE*-ε4 allele and other *APOE* allele variants affect disease risk, which will be valuable to genetic counselling for Alzheimer and other diseases.^22,26^

There is also growing evidence from UK Biobank on the disease risks associated with the 3 main alleles of the *APOE* genotype. For example, ε3ε4 and ε4ε4 genotypes are associated with increased risks of Alzheimer disease, hypercholesterolemia, and ischemic heart disease (IHD), whereas the ε2ε3 allele has been found to be protective against hypercholesterolemia and IHD.^22^ A separate study found that the ε4 genotype is associated with a more pronounced cognitive decline over time in those with late-onset Alzheimer disease.^24^ Machine learning techniques have been used to identify further variants which modulate the *APOE* loci, such as the *SH3BP4* gene, which may protect against the development of Alzheimer disease.^19,27^

The large size of UK Biobank has enabled further insights into the apparent protective effect of rarer variants of the *APOE* haplotype on risk of early-onset Alzheimer disease, even among those with a family history of the disease.^28^ These carriers exhibited significantly lower ApoB levels, suggesting a potential mechanism by which this variant confers protection.^28^ Another study found the *APOE*-ε4 (R251G) and *APOE*-ε3 (V236E) variants to be inversely associated with Alzheimer disease.^29^ These variants influence *APOE*'s lipid-binding region, potentially reducing the levels of insoluble β-amyloid, again suggesting a mechanism through which these variants influence disease risk. A further study identified associations between the *APOE*-ε4 genotype and the *DAB1* gene, which may have significant implications for *DAB1* and *RELN* gene signalling. This highlights their potential role in the production and clearance of β-amyloid, further contributing to our understanding of Alzheimer disease pathology.^22^ Further discoveries will continue to be made regarding the roles and mechanism through which the *APOE* genotype influences the risk of dementia and other conditions.

### Combining Genetics and Imaging Data for Insights Into Neurologic Disease

Pathologic structural and functional characteristics of the brain can be identified in patients with neurologic conditions, such as Alzheimer disease and Parkinson disease, through the use of MRI.^30^ More than 4,000 structural and functional brain imaging-derived phenotypes are readily available from UK Biobank,^31^ and multiple studies have identified associations between specific genetic variants and these phenotypes to uncover new insights regarding the genetic determinants of the brain's function in health and disease.^32,33^ For example, a recent GWAS of phenotypes derived from quantitative susceptibility mapping of the brain MRI scans identified genetic variants associated with biological functions involving iron, calcium, myelin, and extracellular matrix.^34^ The study provided important insights into neurologically relevant brain systems and the genetic blueprints with which they are associated.^35^

The coavailability of imaging and genetic data within UK Biobank allows identification of brain regions and structures vulnerable to specific neurologic disease and genetic determination of suitable targets within these regions. This enables intelligent design of novel pharmaceuticals and increases our understanding of how current drugs work. For example, one study identified white matter–associated genes that were targeted by 79 drugs used to manage psychosis, depression, addiction disorders, Parkinson disease, dementia, and epilepsy, underlying the importance of white matter microstructure in these conditions.^36^ Others have also identified genes which are potential targets for the treatment of neurodegenerative and neuropsychiatric disorders.^37^

### Mendelian Randomization Studies

In recent years, Mendelian randomization (MR) studies have been used to provide evidence of potential causal associations between a wide range of exposures and health-related outcomes.^38^ MR studies are less vulnerable to confounding and other biases that may affect associations in observational epidemiology because the somatic genome is fixed at conception and not influenced by external factors.^38^ MR has been widely used on UK Biobank data.^38^ For example, MR studies have supported the causality of adherence to a Mediterranean diet with a lower risk of dementia^39^ but have refuted the causality of the observed associations between sleep duration or quality and the risk of Alzheimer disease.^40,41^ MR studies have also used the imaging data in UK Biobank to explore the relation between brain imaging-derived phenotypes and Alzheimer disease, stroke, and the risk of major neurodegenerative diseases.^42^ The use of MR has also led to the identification of potential drug targets for multiple sclerosis and stroke.^43,44^

### Blood-Based Biomarkers for Mechanistic and Causal Assessments

The recent release of the metabolomic and proteomic data from UK Biobank has enabled further research into how blood-based biomarkers are related to the risk and detection of neurologic diseases.^9,10^ For example, one study found that plasma levels of glial fibrillary acidic protein and neurofilament light chain, indicators of neuroinflammation and neuronal damage, are strong predictors of the development of dementia up to 15 years prior to diagnosis.^45^ These biomarker data can also help to understand the mechanisms of disease. For example, research using UK Biobank has identified genetic variants associated with regional brain iron accumulation, which was further investigated using the metabolomic and proteomic data to reveal the biological mechanisms linking these rare genetic variants with proteomic indicators of iron transport functions.^34^ Another study combined these biomarkers with brain imaging data to identify an association between periodontal disease and white matter integrity. This evidenced that this association was partly mediated by 2 key plasma proteins, GDF15 and WFDC2, suggesting that poor oral health may increase risk of neurodegenerative disease.^46^

### Risk Prediction

The genetic data contained in UK Biobank have been used to develop polygenic risk scores (PRSs) for a very wide range of phenotypic traits and health outcomes, including Alzheimer's disease and stroke.^47,48^ While PRSs developed in UK Biobank are predominantly restricted to individuals from a European-ancestry (to reduce stratification bias), it is important to note that these often perform less well when applied to African and South Asian populations, owing to differences in allele frequencies and linkage disequilibrium patterns between the ethnic groups. As such, PRSs that include data from non-European ancestries within UK Biobank are now being developed.^49^ Data from the UK Biobank imaging study can also been used for risk prediction, as shown by a study that used resting-state functional brain MRI to identify dementia risk within 9 years, with 80% accuracy.^50^ Future research into the refinement and use of such models may enable more individuals with dementia to be identified earlier, allowing for more effective interventions.

## Limitations

The many advances in neurologic research highlighted in this review demonstrate the strengths of UK Biobank, which include the depth of data (including genetics and blood-based biomarkers on all participants), the ascertainment of a broad range of health outcomes with high diagnostic accuracy over a long duration of follow-up, and the accessibility of the resource to researchers worldwide.

The study was not designed to be representative of the broader population in the United Kingdom and, as such, has limited value in assessing the burden of disease in the population. However, the wide heterogeneity in level of exposures allows the generalizability of associations to be assessed in a range of population subgroups.^8,18^ Replication of analyses in representative cohorts (albeit often substantially smaller in size) has generally found consistent associations.^8^ The proportions of those from minority ethnic groups reflect the distributions in this age group at the time of recruitment in the United Kingdom; however, the ethnic diversity is limited compared with the current UK population. The overall size of the cohort, however, means there are comparatively large numbers of participants from ethnic minority groups^18^; the study currently has the largest collection of WGS data for individuals of South Asian ancestry. This means there is sufficient power to assess associations by ethnicity for at least some conditions. For example, a study that developed a PRS for predicting dementia risk using genetic markers for APOE from UK Biobank participants found that the score was appropriate for predicting dementia for all ethnicities, including South Asian and Black participants (which comprised about 8,000 participants in each group).^51^

Another limitation is that primary care data are currently not available for all participants, meaning that there is reduced power to investigate associations with conditions that are diagnosed and managed largely in primary care. For example, including primary care data for the full cohort would approximately double the number of incident cases of Parkinson disease, when compared with cases ascertained from hospital inpatient admission and death data alone.^52^ It is anticipated that primary care data for the full cohort will become available in the near future.

## Future Directions

UK Biobank plans to continue to enhance the dataset through further blood assays, repeat imaging, and ongoing web-based questionnaires. There are also efforts underway to conduct a “Brain Health Study” to enhance research on neurodegenerative disorders. Participants with neurodegenerative conditions will be invited to undergo various evaluations, including brain MRI, cognitive assessments, blood sampling, and the use of wearable devices to allow researchers to identify disease subtypes and produce more precise health outcome data than is typically available through standard health records. Such comprehensive data will enable investigations into the biological underpinnings of neurodegenerative disease subtypes, potentially guiding disease-specific treatment and prevention approaches. Furthermore, UK Biobank is improving health outcome characterization by linking data with NHS datasets, which include clinical audits and registry information. This integration will also further facilitate research into specific disease subtypes.

## Data Access

UK Biobank data are accessible to all bona fide researchers, regardless of whether they are affiliated with academic, commercial, governmental, or charitable organizations. The data are available for all forms of health-related research that serves the public interest. Researchers must submit an application to access the resource, outlining their research question and the potential public health implications of their proposed project. Applicants from less well-resourced countries, and students from anywhere in the world, are able to access the data at reduced cost. In addition to data, UK Biobank allows applications for biological samples and participant recontact, both subject to stringent review due to the depletable nature of samples and the duty to minimize participant burden. UK Biobank data are only available for analysis on UK Biobank's cloud-based Research Analysis Platform. UK Biobank also provides research credits to students, early career researchers, and researchers from less well-resourced countries to conduct analyses on the platform.

## Conclusions

UK Biobank has become one of the leading resources for health research worldwide and continues to yield invaluable insights into the etiology and prevention of a wide range of diseases. The large scale, extensive genetic and phenotypic data, and long-term follow-up, mean that it is particularly well-suited to research into genetic variations in neurologic disease, and potential approaches to their prevention and treatment. To date, UK Biobank has been used to identify key genetic loci associated with cognitive function and a range of neurologic diseases. The genetic and imaging data have led to significant discoveries regarding the relationship between brain structure, function, and genetic variants. Other genetic and proteomic data have been used to improve risk prediction algorithms for dementia and other neurologic diseases. Each of these data types, alone and in combination, has contributed to the identification of new drug targets and improved understanding of mechanisms of drug action. As UK Biobank continues to be enhanced, and the number of neurologic diseases diagnosed accrues over time, the study will become increasing valuable for both discovery and translational research on genetics and neurologic disease.

## Glossary

APOE: apolipoprotein-E
DXA: dual X-ray absorptiometry
GWAS: genome-wide association studies
IHD: ischemic heart disease
MR: mendelian randomization
NHS: National Health Service
PheWas: phenome-wide association study
PRS: polygenic risk score
SNPs: single-nucleotide polymorphisms
WES: whole-exome sequencing
WGS: whole-genome sequencing
